# Prevalence and Etiology of Community- and Hospital-Acquired Pneumonia in Saudi Arabia and Their Antimicrobial Susceptibility Patterns: A Systematic Review

**DOI:** 10.3390/medicina59040760

**Published:** 2023-04-13

**Authors:** Mohammed Kanan Alshammari, Mzoun Abdulaziz Alotaibi, Ahad Sanad AlOtaibi, Hanan Tareq Alosaime, Mona Awadh Aljuaid, Budur Mohammed Alshehri, Yasmen Bejad AlOtaibi, Asma Ali Alasmari, Ghadi Ali Alasmari, Maram Hussain Mohammed, Shumukh Mohammed Althobaiti, Reem Abdulrahman Almuhaya, Taef Awadh Alkhoshi, Asma Sulayyih Alosaimi, Alanoud Akeel Alotaibi

**Affiliations:** 1 Department of Clinical Pharmacy, King Fahad Medical City, Riyadh 12211, Saudi Arabia; 2 College of Medicine, Qassim University, Buraydah 51411, Saudi Arabia; 3 Department of Pharmacy, Taif University, Taif 26311, Saudi Arabia; 4 Clinical Pharmacy & Pharmacology Department, Ibn Sina National College for Medical Studies, Jeddah 22421, Saudi Arabia; 5 Department of Ambulatory Care Pharmacy, International Medical Center, Jeddah 23214, Saudi Arabia; 6 College of Pharmacy, King Khalid University, Abha 62529, Saudi Arabia; 7 Prince Sultan Military Medical City, Riyadh 11452, Saudi Arabia; 8 Department of Nursing, King Saud Bin Abdulaziz University, Riyadh 11461, Saudi Arabia

**Keywords:** prevalence, etiology, community-acquired pneumonia, hospital-acquired pneumonia, Saudi Arabia, antimicrobial susceptibility

## Abstract

(1) *Background and Objectives*: Pneumonia is a major cause of morbidity and mortality worldwide, including in Saudi Arabia, and the prevalence and etiology of the disease varies depending on the setting. The development of effective strategies can help reduce the adverse impact of this disease. Therefore, this systematic review was conducted to explore the prevalence and etiology of community-acquired and hospital-acquired pneumonia in Saudi Arabia, as well as their antimicrobial susceptibility. (2) *Materials and Methods*: The Preferred Reporting Items for Systematic Reviews and Meta-Analyses (PRISMA) 2020 recommendations were followed for this systematic review. Several databases were used to perform a thorough literature search, and papers were then assessed for eligibility by two independent reviewers. The Newcastle-Ottawa Scale (NOS) was used to extract the data from the relevant research and evaluate its quality. (3) *Results*: This systematic review included 28 studies that highlighted the fact that gram-negative bacteria, particularly *Acinetobacter* spp. and *Pseudomonas aeruginosa*, were the common cause of hospital-acquired pneumonia, while *Staphylococcus aureus* and *Streptococcus* spp. were responsible for community-acquired pneumonia in children. The study also found that bacterial isolates responsible for pneumonia showed high resistance rates against several antibiotics, including cephalosporins and carbapenems. (4) *Conclusions*: In conclusion, the study found that different bacteria are responsible for community- and hospital-acquired pneumonia in Saudi Arabia. Antibiotic resistance rates were high for several commonly used antibiotics, highlighting the need for rational antibiotic use to prevent further resistance. Moreover, there is a need to conduct more regular multicenter studies to assess etiology, resistance, and susceptibility patterns of pneumonia-causing pathogens in Saudi Arabia.

## 1. Introduction

Pneumonia is an inflammation of the parenchyma cells of the lungs caused by an infectious agent, either a gram-positive or gram-negative microbe. It is a major cause of morbidity and mortality worldwide, particularly in vulnerable populations such as the elderly, immunocompromised individuals, and those with underlying health conditions [1]. This serious respiratory infection can be acquired in both community and hospital settings [2]. According to the Infectious Diseases Society of America (IDSA), community-acquired pneumonia (CAP) is an acute infection of the pulmonary tissues in a patient who did not contract it from a healthcare system or within the first 48 h after being hospitalized. Risk factors for CAP include old age, alcohol consumption, smoking, previous history of pneumonia, viral respiratory infections, diabetes, chronic obstructive pulmonary disease, and immunosuppression [3,4]. CAP is a significant contributor to morbidity and death on a global scale [5]. According to reports, CAP kills between 1.6 to 10.6 persons per year in Europe and an estimated 500,000 adults annually in Asia [3,6,7]. Adult CAP patient death rates in Africa range from 6% to 15%. In Sub-Saharan Africa, there are similarly substantial rates of morbidity and mortality, with 4 million cases of pneumonia reported there each year and 200,000 fatalities [8,9,10]. The frequency of bacterial CAP in Ethiopia varies between 38.7% and 45% [10,11]. Whereas, hospital-acquired pneumonia (HAP), also known as nosocomial pneumonia, is a life threatening lower respiratory infection that does not incubate at the time of hospital admission, but manifests clinically two or more days later [2]. The risk factors for HAP include prolonged hospitalization, mechanical ventilation, immunosuppression, and the use of antibiotics. The antimicrobial susceptibility of CAP and HAP varies depending on the etiologic agent [12]. According to the World Health Organization (WHO), hospital-acquired infections (HAIs) are responsible for an estimated 1.4 million deaths worldwide each year, with pneumonia being one of the most common types of HAI [13]. However, it is difficult to determine the exact mortality rate of HAP because it can vary widely depending on a number of factors, including the patient population, the severity of the pneumonia, and the availability and effectiveness of treatment options. It is also worth noting that the mortality rate of HAP can vary widely depending on the region and hospital setting. A systematic review and meta-analysis published in 2016 by Torres et al. found that the overall mortality rate of HAP in Europe was 17%, while the rate was higher in the United States (21%) and lower in Asia (13%). The study also found that the mortality rate was higher in patients with comorbidities, such as diabetes and chronic kidney disease, and in patients with severe pneumonia [14].

As in other countries, pneumonia is a major cause of morbidity and mortality in Saudi Arabia. The prevalence and etiology of the disease varies depending on the setting, with an estimated incidence of 1.5 to 2.5 cases per 1000 people per year of CAP, and 5 to 10 cases per 1000 people per year of HAP. A study published in 2018 by Al-Ghamdi et al. [2] in Saudi Arabia found that the mortality rate of HAP among patients in an intensive care unit (ICU) was 26.4%. The study involved a retrospective analysis of medical records of patients admitted to the ICU at a tertiary care hospital in Saudi Arabia between January 2012 and December 2016. The study found that factors such as older age, chronic obstructive pulmonary disease (COPD), and the presence of sepsis were associated with a higher risk of mortality in patients with HAP. Another study conducted by Balkhair et al. [3] in Saudi Arabia in 2016 found that the overall mortality rate of HAP among hospitalized patients was 32.4%. This study was a retrospective analysis of medical records of patients admitted to two hospitals in Saudi Arabia between January 2012 and December 2013. The study found that the mortality rate was higher in patients with comorbidities such as diabetes, cardiovascular disease, and COPD. 

In many countries, where the risk of pneumonia is high, excessive irrational use of antibiotics is leading to the emergence of antimicrobial resistance (AMR), limiting the use of previously available antibiotics and pushing to newer, more expensive medications [15,16]. Both gram-positive and gram-negative bacteria are more likely to exhibit AMR towards first- and second-line antibiotics used to treat CAP and HAP [17,18,19]. Although more modern antimicrobial medications, including omadacycline, delafloxacin, and lefamulin have recently received approval for pneumonia, there is not sufficient data to recommend using them as first-line medications [20]. These multi-drug resistance (MDR) infections have increased the need for routine antimicrobial susceptibility testing to identify the preferred antibiotics, and to rationalize the use of antibiotics by implementing antimicrobial stewardship programs [15,21]. Overall, the mortality rate of HAP is a significant concern for healthcare providers and policymakers, and efforts are needed to prevent and manage this serious infection. The development of effective prevention and treatment strategies, as well as the implementation of infection control measures, can help reduce the incidence and mortality rate of CAP and HAP in both Saudi Arabia and worldwide. Therefore, this systematic review was conducted to explore the prevalence and etiology of CAP and HAP in Saudi Arabia, as well as their antimicrobial susceptibility.

## 2. Materials and Methods

### 2.1. Protocol Design

This study was conducted following the Preferred Reporting Items for Systematic Reviews and Meta-Analysis (PRISMA) guidelines 2020 [22].

### 2.2. Identification of the Research Question

The clear and specific research question was identified focusing on the prevalence and etiology of CAP and HAP and as their antimicrobial susceptibility in Saudi Arabia. This question is important because it addresses a significant public health concern, and can help guide healthcare providers in the appropriate management of pneumonia. By identifying the common pathogens responsible for pneumonia and their antimicrobial resistance patterns, healthcare providers can make informed decisions regarding the choice of antibiotics and prevent the further development of antibiotic resistance. The specificity of the research question allows for a focused and targeted search of the literature, which can help ensure that the study is relevant and informative. Overall, identifying a clear and specific research question is a crucial step in conducting a meaningful and impactful study.

### 2.3. Eligibility Criteria

The inclusion criteria were studies conducted in Saudi Arabia, studies that report the prevalence, etiology, and/or antimicrobial susceptibility patterns of CAP and/or HAP, studies published in the English language, and studies published between 2000 and onward. Whereas, the research papers that do not meet the inclusion criteria, or those that do not provide information on the prevalence and etiology of CAP and HAP and as their antimicrobial susceptibility in Saudi Arabia, were excluded.

### 2.4. Information Sources

A comprehensive literature search was performed using multiple databases. PubMed, Scopus, Google Scholar, Science Direct, Embase, and the Cochrane Library were searched to identify relevant studies. Additionally, reference lists of identified studies were also hand-searched. This approach of using multiple databases and hand-searching reference lists helped ensure that the authors included all relevant studies in their systematic review.

### 2.5. Search Strategy

An inclusive search strategy was developed using relevant keywords and medical subject heading (MeSH) terms related to the topic. The search strategy was designed for each database and was reviewed by a second researcher. These databases were searched with the following key terms: “prevalence”, “etiology”, “community-acquired pneumonia”, “hospital-acquired pneumonia”, “healthcare-associated pneumonia”, “antimicrobial”, “antibiotics”, “susceptibility”, “resistance”, “Saudi Arabia” and related MeSH terms with “AND” or “OR”.

### 2.6. Study Selection

Two researchers independently screened the titles and abstracts of all identified studies against the eligibility criteria. Full-text articles of potentially eligible studies were retrieved and reviewed for eligibility. After evaluating all databases, studies were screened for duplication detection, which were then deleted, respectively. The articles were excluded after screening the title and abstract. Review articles and book chapters were also excluded. Any disagreements between the two researchers were resolved through discussion and consensus.

### 2.7. Data Extraction

Data were extracted from the included studies using a standardized data extraction form. The following data were extracted: study characteristics, authors, year of publication, study design, study population characteristics, sample size, study setting, study period, population type, etiology of CAP and/or HAP, prevalence of infection, microbiological tests used for diagnosis, antimicrobial susceptibility patterns, isolated pathogens, and quality assessment.

### 2.8. Quality Assessment

The quality of the included studies was assessed using the Newcastle-Ottawa Scale (NOS). The NOS is a tool used to assess the quality of non-randomized studies such as cohort or case-control studies. It evaluates the quality of the studies based on three main categories: selection of the study groups, comparability of the groups, and ascertainment of the outcome of interest. Two researchers independently assessed the quality of each included study. Any discrepancies were resolved through discussion and consensus. Assessing the quality of the included studies is an important step in a systematic review, as it helps to ensure that the results are reliable and valid. The use of a standardized tool such as the NOS helps to minimize bias and subjectivity in the quality assessment process.

### 2.9. Data Synthesis and Reporting

A narrative synthesis of the data was conducted. The findings were presented in tables and figures, and a summary of the findings was provided according to the PRISMA 2020 guidelines. Software including Microsoft excel 365 (Redmond, WA, USA) and Endnote 20 (Philadelphia, PA, USA) were used for this systematic review. 

## 3. Results

### 3.1. Study Selection

**Figure 1:**
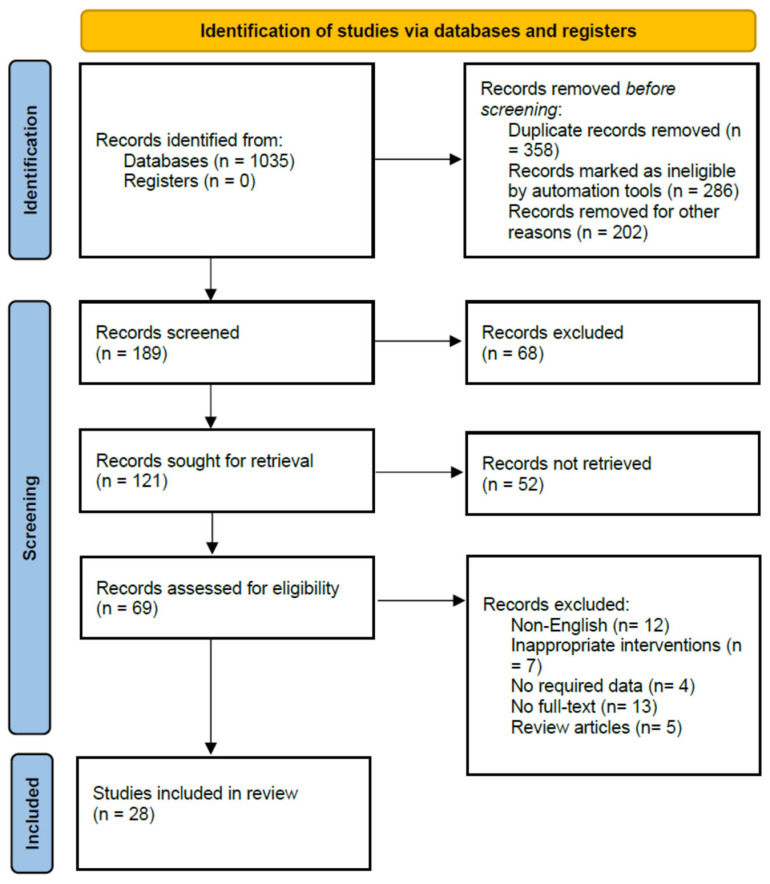
Flowchart of included studies.

Initially, 1035 studies reporting the prevalence of pneumonia were identified using electronic searches (through databases searching and other sources). A total of 189 articles remained after the removal of duplicates and for other reasons. The titles and abstracts were then screened, and 68 were excluded which did not meet eligibility criteria. The remaining 41 studies were excluded due to the following reasons: Non-English (*n* = 12), inappropriate interventions (*n* = 7), No full-text (*n* = 13), no required data (*n* = 4), and review articles (*n* = 5). Finally, 28 studies met the eligibility criteria and were therefore included in this systematic review (Figure 1).

### 3.2. Characteristics of Included Studies

The characteristics of 28 included studies are depicted in Table 1 [23,24,25,26,27,28,29,30,31,32,33,34,35,36,37,38,39,40,41,42,43,44,45,46,47,48,49,50], of which 14 included studies were retrospective. More than half of the included studies were published between 2010 and 2020. A total of 20,899 study participants were included to assess the prevalence of pneumonia and its etiology. The smallest sample size was 32 [30], whereas the largest sample size was 4192 [43]. A total of 15 studies were conducted on adults, while only four studies were conducted on children [33,36,46,48]. The majority of the studies reported the prevalence of CAP and HAP. 

Pneumonia is mainly caused by gram-negative bacteria. The gram-negative bacteria include *Acinetobacter* spp., *Pseudomonas aeruginosa*, and *Klebsiella* spp. *Acinetobacter* spp. and *Pseudomonas aeruginosa* were the common cause of VAP, while *S. pneumoniae* and *Klebsiella* spp. were responsible for CAP. In children, *Staphylococcus aureus* was the major cause of CAP, followed by *Klebsiella pneumoniae* and *Streptococcus* spp. [46]. Other pathogens such as *Pseudomonas* spp., *H. influenzae*, *M. catarrhalis*, and MRSA had a marginal role in causing pneumonia [23].

### 3.3. Antimicrobial Susceptibility/Resistance Pattern

Table 2 summarizes the susceptibility and resistance pattern of bacterial isolates responsible for pneumonia. The disc diffusion method was used in the majority of the studies to test the susceptibility/resistance patterns of antibiotics [23,24,29,30,32,34,43]. The susceptibility and resistance were determined against different classes of antibiotics: carbapenems (meropenem and imipenem), penicillins (penicillin, amoxicillin, piperacillin, and ampicillin), quinolones (ciprofloxacin and levofloxacin), cephalosporins (cefuroxime, ceftriaxone, cefotaxime, cefixime, cefepime, and ceftazidime), aminoglycosides (amikacin and gentamicin), folate pathway inhibitors (sulfamethoxazole/trimethoprim), polymixin (colistin), and macrolides (azithromycin). All included studies interpreted the results according to the guidelines of the Clinical and Laboratory Standards Institute (CLSI). The results obtained reported that the pathogens varied in their susceptibility and resistance rates to the antibiotics used for the treatment of pneumonia.

*Acinetobacter* spp. showed a high resistance rate to quinolones (ciprofloxacin and moxifloxacin), cephalosporins (ceftazidime, and cefepime), and carbapenems (meropenem and imipenem); these species are highly susceptible to colistin [32,34]. However, two of the included studies reported that meropenem exhibit high susceptibility rates against *Acinetobacter* specie [24,47]. Similarly, most of the studies reported that *P. aeruginosa* is resistant to carbapenems and cephalosporins, but is susceptible to levofloxacin, amikacin and gentamicin [40,43,51]. However, Balkhy and his colleagues documented that gentamicin showed 100% resistance against *P. aeruginosa* [24]. Moreover, *Klebsiella* spp. and *S. pneumoniae* showed high resistance rates against penicillins (penicillin and ampicillin) [23,27,40,47]. Resistance rates had also been observed in antibiotics azithromycin, aztreonam, cephalothin, and fosfomycin [38,40,45,50].

### 3.4. Quality Assessment

According to NOS, the quality of each study was assessed on the basis of three-dimensional criteria that included: (1) selection, (2) comparability, and (3) outcomes. Each study was scored using a scale with a maximum of nine stars. Based on scores, the study scored ≥7 was considered high quality, 4–6 medium quality, and 0–4 low quality. The stars that were awarded to studies ranged from six to eight, and the average score was 7.4 (Table 3).

## 4. Discussion

Pneumonia is a widespread respiratory infection that affects people of all ages, although it is more common in young children and the elderly [52]. According to the World Health Organization (WHO), pneumonia is one of the leading causes of death in children under the age of five, accounting for approximately 15% of all deaths in this age group [53]. In 2019, there were an estimated 2.5 million deaths from pneumonia worldwide, with most of these occurring in low- and middle-income countries [54]. The prevalence of pneumonia varies depending on the region and the population. In developed countries, pneumonia is more common during the winter months, with a higher incidence in older adults and people with underlying health conditions [55,56]. However, in developing countries, pneumonia is more prevalent year-round. Children under the age of five are particularly vulnerable, often due to poor nutrition, lack of access to healthcare, and living in crowded conditions [57].

This systematic review aimed to present a comprehensive summary of all relevant published data regarding pneumonia in Saudi Arabia. Most of the studies reported a high prevalence rate of HAP in patients admitted to ICU [32,33,35]. The prevalence of ventilator-associated pneumonia (VAP) in Saudi Arabia is a significant concern, as it is one of the most common healthcare-associated infections in the country. The variation in the prevalence of the VAP found among prospective and retrospective studies is likely due to the selection criteria of the healthcare setting and patient population [34,39,43]. Most of the studies included patients with VAP only. Moreover, the nutritional status, diagnostic criteria and resource availability can be the source of differences in the prevalence of VAP [58]. One study conducted in a tertiary care hospital in Riyadh reported a VAP incidence rate of 15.4%, which is higher than the global average of 7–8% [39]. Another study in a pediatric intensive care unit in Jeddah reported a VAP rate of 10.2% [33]. However, the pediatric studies all around the world reported the VAP prevalence of 2–35% [58,59,60,61]. The most common pathogens responsible for VAP include gram-negative bacteria such as *Acinetobacter baumannii*, *Pseudomonas aeruginosa*, and *Klebsiella pneumoniae*, as well as gram-positive bacteria such as *Staphylococcus aureus* and *Streptococcus pneumoniae*. This finding could demonstrate the endemicity of pathogens in Saudi Arabia. Similarly, these pathogens are also responsible for VAP in Asian countries [62]. The high prevalence of VAP in Saudi Arabia is attributed to several factors, including the overuse of antibiotics, inadequate infection control measures, and the use of invasive procedures such as mechanical ventilation [51]. 

Several studies reported that CAP is one of the leading infectious diseases worldwide [27,28]. The global incidence of CAP is estimated to be 450 million cases per year, with a mortality rate of 5–10% [63]. The etiology of CAP can be attributed to a variety of pathogens, including bacteria, viruses, and fungi. *Streptococcus pneumoniae* and *Klebsiella* spp. are the most common bacterial cause of CAP, accounting for approximately 26–80% of cases [28,46]. The etiology of CAP in Saudi Arabia is similar to that in other regions, with *Streptococcus pneumoniae* being the most common pathogen responsible for CAP. The *Streptococcus* species are challenging pathogens that are responsible for CAP due to their intrinsic resistance to antibiotics [23]. However, in Asian counties, *Klebsiella pneumoniae* is the most common pathogen responsible for CAP [64]. The prevalence and etiology of CAP can vary depending on factors such as age, underlying health conditions, and geographic location. Older adults and individuals with chronic health conditions, such as diabetes and COPD, are at higher risk of developing CAP [65,66]. In Saudi Arabia, the prevalence of CAP is not well documented due to limited epidemiological data. However, some studies suggest that CAP is a significant health burden in the country, particularly in the adult population [29,31,42]. Other common pathogens include Haemophilus influenzae, Mycoplasma pneumoniae, and *M. catarrhalis*. Viral causes of CAP, such as influenza and respiratory syncytial virus (RSV), have also been reported in some cases [23,26,29]. Certain risk factors for CAP have been identified in Saudi Arabia, including smoking, COPD, diabetes mellitus, and immunosuppression [67]. These risk factors are associated with an increased prevalence of CAP. 

Different guidelines suggested that VAP and CAP can be treated with a wide range of antibiotics. However, concerns exist about side effects and selective pressure for resistance to antibiotics. The resistance of most of the bacterial isolates is due to the inherent resistance of the pathogens. For instance, aminopenicillin (ampicillin, amoxicillin, and penicillin) showed resistance against *K. pneumoniae* and *Streptococcus* spp. [27,29,40,47,49]. Similarly, other studies conducted in Iran and Ethiopia reported that *Streptococcus* spp. are highly resistant (>50%) to penicillin and its derivatives [68]. However, the susceptibility to quinolones was up to 90%, whereas the study covering eastern, central and western regions of Saudi Arabia reported the susceptibility up to 98% [23,27]. A. baumannii is resistant to cephalosporins and quinolones, and some bacterial isolates are resistant to carbapenems. The rate of resistance to cephalosporins in Saudi Arabia ranges from 9–98% [24,47]. However, the majority of the strains are susceptible to colistin [34,45,51]. Current data on antibiotic resistance and susceptibility patterns is insufficient, and there are calls for more comprehensive studies to be conducted, including testing for atypical pathogens. The hallmark of the treatment of pneumonia is the use of newer antibiotics, and care must be taken to avoid the development of microbial resistance in the general population against efficacious antibiotics.

Studies reported from gulf countries documented adherence to Gulf Cooperation Council (GCC) guidelines for the treatment of pneumonia [69,70]. The collection of representative national data will assist healthcare officials in making public health decisions. Preventing pneumonia in Saudi Arabia requires a multi-faceted approach, including strict adherence to infection control protocols, vaccination against *S. pneumoniae* and *H. influenzae*, appropriate use of antibiotics, and early identification and management of patients at risk of disease. The Saudi Ministry of Health has implemented several initiatives to reduce the incidence of respiratory tract infections, including the development of guidelines for infection prevention and management, mandatory reporting of pneumonia cases, and increased surveillance of healthcare-associated infections. Prevention strategies for pneumonia in Saudi Arabia include vaccination against *S. pneumoniae* and influenza, as well as education on hand hygiene and respiratory etiquette. Treatment of pneumonia typically involves antibiotics targeted at the identified pathogen and supportive care, including oxygen therapy and hydration [71]. 

## 5. Conclusions

*Acinetobacter* spp. and *Pseudomonas aeruginosa* were the common cause of VAP, while *S. pneumoniae* and *Klebsiella* spp. were responsible for CAP in Saudi Arabia. However, in children, *Staphylococcus aureus* was the major cause of CAP, followed by *Klebsiella pneumoniae* and *Streptococcus* species. The bacterial isolates exhibited good susceptibility to colistin, while the majority of isolates demonstrated high resistance rates to aminopenicillin, third-generation cephalosporins, quinolones, and aminopenicillin. Rational use of antibiotics should be taken into consideration in order to prevent further antibiotic resistance. Moreover, more multicenter studies, including in all regions of Saudi Arabia, are required to explore the distribution of various pathogens responsible for pneumonia. Regular assessment of the isolates’ etiology resistance and susceptibility pattern is also recommended. 

## Figures and Tables

**Table 1:** Characteristics of eligible studies conducted in Saudi Arabia.

Author and Year	Study Setting	Study Period	Sample Size	Population Type	Study Design	Prevalence of Infection	Top 3 Isolated Pathogens		
Batool et al., 2021 [23]	1 hospital	May 2019–October 2019	165	Adults	Cross-sectional study	CAP (46.6%)	*S. pneumoniae* (34%)	*H. influenzae* (16%)	*S. aureus* (30%)
Balkhy et al., 2014 [24]	ICU	October 2004–June 2009	248	Adults	Retrospective study	VAP (100%)	*Acinetobacter* spp. (35.1%)	*P. aeruginosa* (25.4%)	*S. aureus* (17.3%)
Farahat et al., 2021 [25]	1 hospital	2016 -2019	218	Adults	Retrospective study	CAP (100%)	*S. aureus* (2.5%)	MRSA (2.3%)	*Klebsiella* spp. (1.3%)
Rahman et al., 2000 [26]	5 medical centers	January–July	129	-	Retrospective study	LRTIs (100%)	*H. influenzae* (100%)	-	-
Memish et al., 2004 [27]	3 hospitals	January 2000–December 2000	154	Mixed	Cross-sectional study	CAP (100%)	*S. pneumoniae* (100%)	-	-
Alshahwan et al., 2019 [28]	Pulmonary department	2010–2017	800	Adults	Retrospective study	CAP (80%)	-	-	-
						HAP (20%)			
Akbar et al., 2001 [29]	1 hospital	January 1998–December 1999	354	Adults	Prospective study	CAP (24%)	*H. influenzae* (51.7%)	*S. aureus* (14.1%)	*M. catarrhalis* (11.7%)
						HAP (76%)	*Pseudomonas* spp. (27.1%)	*S. aureus* (15.2%)	*H. influenzae* (14.4%)
Babay et al., 2000 [30]	1 hospital	July 1998–December 1999	32	Mixed	Retrospective study	Pneumonia (21.8%)	*M. catarrhalis* (100%)	-	-
Albarak et al., 2018 [31]	13 hospitals	August 2016–September 2016	266	Adults	Prospective case series	CAP (100%)	*S. pneumoniae* (18%)	-	-
Obeid et al., 2015 [32]	1 hospital	2006, 2009 and 2012	1952	-	Retrospective study	VAP (100%)	*A. Baumannii* (100%)	-	-
Almuneef et al., 2004 [33]	ICU	May 2000–December 2002	361	Children	Prospective study	VAP (10.2%)	*P. aeruginosa* (56.8%)	*S. aureus* (18.9%)	*K. pneumoniae* (10.8%)
Saleem et al., 2022 [34]	ICU	2019–2022	591	Adults	Prospective study	VAP (27.5%)	*A. baumannii* (21.4%)	-	-
El-Saed et al., 2013 [35]	ICU	August 2003–June 2009	457	Adults	Prospective study	VAP (100%)	*Acinetobacter* spp. (26.5%)	*P. aeruginosa* (21.7%)	*S. aureus* (15.3%)
Osman et al., 2020 [36]	PICU	January 2015–March 2018	141	Children	Prospective study	VAP (100%)	*P. aeruginosa* (34.0%)	*K. pneumoniae* (18.1%)	*S. maltophilia* (13.6%)
Kabrah et al., 2021 [37]	ICU	November 2020–January 2021	96	Adults	Retrospective study	LRTIs (53.1%)	*P. aeruginosa* (66.7%)	*A. baumannii* (13.7%)	*K. oxytica* (4.0%)
Ibrahim et al., 2018 [38]	ICU	December 2016–January 2018	3736	Adults	Retrospective study	9.6%	*Acinetobacter* spp. (34.6%)	*P. aeruginosa* (24.5%)	*K. pneumoniae* (14.9%)
Al-Dorzi et al., 2012 [39]	ICU	August 2003–June 2009	2812	Adults	Prospective study	VAP (15.4%)	-	-	-
Saleem et al., 2023 [40]	ICU	January 2019–December 2019	591	-	Prospective study	VAP (43%)	*K. pneumoniae* (24%)	*A. baumannii* (21.5%)	*P. aeruginosa* (15.3%)
Othman et al., 2017 [41]	ICU	September 2012–August 2013	48	Adults	Prospective study	VAP (35.4%)	*P. aeruginosa* (41.1%)	*S. aureus* (17.6%)	*K. pneumoniae* (11.7%)
Balkhy et al., 2006 [42]	1 hospital	May 2003	562	Adults	PPS	CAP (34.9%)	*P. aeruginosa* (21.3%)	*Klebsiella* spp. (10.1%)	*Pseudomonas* spp. (7.9%)
Al-Johani et al., 2010 [43]	ICU	January 2004–June 2009	4192	Adults	Retrospective study	VAP (43%)	*Acinetobacter* spp. (31.7%)	*P. aeruginosa* (30.6%)	*E. coli* (14.0%)
Mwanri et al., 2014 [44]	ICU	2010–2012	496	Mixed	Retrospective study	VAP (14.8%)	*Acinetobacter* spp. (57.4%)	*Klebsiella* ESBL (13.2%)	MRSA (9.8%)
Bshabshe et al., 2016 [45]	ICU	2014–2015	105	Adults	Observational study	100%	*A. baumannii* (46.6%)	*A. haemolyticus* (30.4%)	A. complex (18.0%)
Zaki et al., 2021 [46]	1 hospital	January 2017–December 2019	163	Children	Retrospective study	CAP (26.4%)	*S. aureus* (37.2%)	*K. pneumoniae* (30.2%)	*Streptococcus* spp. (14%)
Hakami et al., 2022 [47]	1 hospital	May 2016–December 2019	1151	Mixed	Cross-sectional study	49.3%	*P. aeruginosa* (28.5%)	K. pneumoniae (17.6%)	*A. baumannii* (15.1%)
Walid et al., 2016 [48]	1 hospital	January 2019–Jan 2015	122	Children	Retrospective study	Pneumonia (26.2%)	*P. aeruginosa* (11.5%)	*S. pneumoniae* (7.6%)	*S. aureus* (7.6%)
Al-Munjem et al., 2022 [49]	1 medical center	January 2016–December 2017	405	Mixed	Retrospective study	Pneumonia (47.4%)	*S. pneumoniae* (90.9%)	*P. aeruginosa* (84.1%)	*K. pneumoniae* (84.1%)
Marie et al., 2010 [50]	1 hospital	-	552	Mixed	Retrospective study	Pneumonia (49.4%)	*M. pneumoniae* (42.2%)	-	-

CAP = Community-acquired pneumonia, ESBL = Extended Spectrum Beta Lactamase, HAP = hospital-acquired pneumonia, ICU = Intensive Care Unit, MRSA = Methicillin Resistant *Staphylococcus aurous*, VAP = ventilator-acquired pneumonia.

**Table 2:** Susceptibility and resistance patterns of isolates.

Author and Year	Isolated Organism	Top 3 Most Sensitive Antibitoics			Top 3 Most Resistant Antibiotic		
Batool et al., 2021 [23]	*S. pneumoniae*	Amoxicillin/Sulbactam (96%)	Ceftriaxone (92%)	Amikacin (92%)	Co-trimoxazole (43%)	Ampicillin (39%)	Ciprofloxacin (39%)
	*H. influenzae*	Levofloxacin (91%)	Ciprofloxacin (83%)	Amikacin (83%)	Doxycycline (59%)	Clarithromycin (59%)	Azithromycin (59%)
	*S. aureus*	Amikacin (90%)	Ceftriaxone (80%)	Ampicillin (80%)	Co-trimoxazole (60%)	Clarithromycin (40%)	Doxycycline (40%)
Balkhy et al., 2014 [24]	*Acinetobacter* spp.	Meropenem (25%)	Piperacillin/Tazobactam (17%)	Amikacin (11%)	Imipenem (95%)	Ciprofloxacin (98%)	Gentamicin (97%)
	*P. aeruginosa*	Amikacin (68%)	Meropenem (43%)	Piperacillin/Tazobactam (8%)	Gentamicin (100%)	Cefepime (98%)	Ciprofloxacin (97%)
	*S. aureus*	Gentamicin (56%)	Ciprofloxacin (35%)	Penicillin (37%)	Oxacillin (100%)	Vancomycin (100%)	Erythromycin (88%)
Farahat et al., 2021 [25]	-	-	-	-	-	-	-
Rahman et al., 2000 [26]	*H. influenzae*	Ciprofloxacin (100%)	Ceftazidime (100%)	Amoxiclav (98%)	Ampicillin (13.2%)	Tetracycline (7%)	Chloramphenicol (5%)
Memish et al., 2004 [27]	*S. pneumoniae*	Vancomycin (100%)	Levofloxacin (98%)	Ceftriaxone (85%)	Erythromycin (15%)	Penicillin (14%)	Sulfamethoxazole/trimethoprim (9%)
Alshahwan et al., 2019 [28]	*-*	-	-	-	-	-	-
Akbar et al., 2001 For CAP [29]	*H. influenzae*	Cefuroxime (97%)	Ciprofloxacin (84%)	Ceftriaxone (87%)	Vancomycin (97%)	Gentamicin (97%)	Imipenem (94%)
	*S. pneumoniae*	Co-amoxiclav (100%)	Cefuroxime (100%)	Erythromycin (100%)	Penicillin (67%)	Gentamicin (67%)	Aztreonam (67%)
	*M. Catarrhalis*	Co-amoxiclav (100%)	Cefuroxime (100%)	Erythromycin (100%)	Penicillin (87%)	Ceftriaxone (87%)	Erythromycin (14%)
Akbar et al., 2001 For HAP [29]	*Pseudomonas* spp.	Amikacin (85%)	Gentamicin (84%)	Piperacillin (70%)	Erythromycin (98%)	Co-amoxiclav (93%)	Ampicillin (93%)
	*S. aureus*	Vancomycin (86%)	Oxacillin (81%)	Erythromycin (67%)	Ampicillin (86%)	Co-amoxiclav (86%)	Ciprofloxacin (86%)
	*Enterobacter* spp.	Imipenem (100%)	Amikacin (100%)	Ciprofloxacin (79%)	Ampicillin (93%)	Co-amoxiclav (93%)	Cefuroxime (86%)
Babay et al., 2000 [30]	*M. catarrhalis*	Ciprofloxacin (-)	Gentamicin (-)	Tetracycline (-)	-	-	-
Albarrak et al., 2018 [31]	*-*	-	-	-	-	-	-
Obeid et al., 2015 [32]	*A. baumannii*	Colistin (-)	Gentamicin (-)	Tigecycline (-)	Amikacin (-)	Ceftazidime (-)	Ciprofloxacin (-)
Almuneef et al., 2004 [33]	*-*	-	-	-	-	-	-
Saleem et al., 2022 [34]	*A. baumannii*	Colistin (100%)	Amikacin (48%)	Gentamicin (17%)	Co-amoxiclav (-)	Ciprofloxacin (-)	Ampicillin (-)
Al-Saed et al., 2013 [35]	-	-	-	-	-	-	-
Osman et al., 2020 [36]	-	-	-	-	-	-	-
Kabrah et al., 2018 [37]	Overall	Teicoplanin (100%)	Vancomycin (100%)	Synercid (100%)	Penicillin (100%)	Aztreonam (96%)	Cefotaxime (88%)
Ibrahim et al., 2018 [38]	*Acinetobacter* spp.	Colistin (96%)	Sulfamethoxazole/Trimethoprim (7%)	Gentamicin (4%)	Aztreonam (97%)	Cefepime (97%)	Ceftazidime (97%)
	*P. aeruginosa*	Amikacin (82%)	Tobramycin (80%)	Gentamicin (69%)	Cefuroxime (66%)	Cefotaxime (63%)	Cefepime (53%)
	*K. pneumoniae*	Colistin (100%)	Imipenem (59%)	Amikacin (54%)	Cefuroxime (71%)	Sulfamethoxazole/Trimethoprim (71%)	Ceftazidime (67%)
Al-Dorzi et al., 2012 [39]	*-*	-	-	-	-	-	-
Saleem et al., 2023 [40]	*K. pneumoniae*	Colistin (59%)	Piperacillin/Tazobactam (52%)	Gentamicin (52%)	Ampicillin (92%)	Levofloxacin (92%)	Amoxiclav (89%)
	*A. baumannii*	Tigecycline (40%)	Piperacillin/Tazobactam (26%)	Gentamicin (20%)	Amoxiclav (100%)	Ciprofloxacin (100%)	Meropenem (100%)
	*P. aeruginosa*	Tigecycline (96%)	Colistin (72%)	Amikacin (72%)	Amoxiclav (84%)	Cefuroxime (84%)	Cephalothin (84%)
Othman et al., 2017 [41]	-	-	-	-	-	-	-
Balkhy et al., 2006 [42]	-	-	-	-	-	-	-
Al-Johani et al., 2010 [43]	*Acinetobacter* spp.	Imipenem (10%)	Meropenem (10%)	Ciprofloxacin (10%)	Cefepime (-)	Ciprofloxacin (-)	Ceftazidime (-)
	*P. aeruginosa*	Ciprofloxacin (49%)	Ceftazidime (44%)	Carbapenems (26%)	Meropenem (-)	Meropenem (-)	Cefepime (-)
	*E.coli*	Cefepime (50%)	Ceftazidime (46%)	Cefotaxime (46%)	Ampicillin (-)	Sulfamethoxazole/Trimethoprim (-)	Ciprofloxacin (-)
Mwanri et al., 2014 [44]	-	-	-	-	-	-	-
Bshabshe et al., 2016 [45]	*A. baumannii*	Colistin (100%)	Sulfamethoxazole/Trimethoprim (74%)	Amikacin (16%)	Moxifloxacin (100%)	Meropenem (100%)	Fosfomycin (100%)
	*A. haemolyticus*	Colistin (100%)	Sulfamethoxazole/Trimethoprim (68%)	Amikacin (18%)	Ampicillin (100%)	Moxifloxacin (100%)	Fosfomycin (100%)
	A. complex	Colistin (100%)	Rifampicin (100%)	Sulfamethoxazole/Trimethoprim (78%)	Amikacin (100%)	Ciprofloxacin (100%)	Imipenem (100%)
Zaki et al., 2021 [46]	*-*	-	-	-	-	-	-
Hakami et al., 2022 [47]	*P. aeruginosa*	Cefepime (99%)	Tigecycline (99%)	Imipenem (98%)	Piperacillin/Tazobactam (51%)	Ciprofloxacin (25%)	Ampicillin (10%)
	*K. pneumoniae*	Amoxicillin (99%)	-	-	Ampicillin (82%)	Ceftriaxone (9%)	Piperacillin/Tazobactam (7%)
	*A. baumannii*	Meropenem (99%)	Ceftriaxone (99%)	Amoxiclav (98%)	Piperacillin/Tazobactam (52%)	Ampicillin (38%)	Ciprofloxacin (5%)
Walid et al., 2016 [48]	Overall	-	-	-	Ceftriaxone (24%)	Cefuroxime (21%)	Cefotaxime (3%)
Al Munjem et al., 2022 [49]	*S. pneumoniae*	Levofloxacin (67%)	Oxacillin (52%)	Ciprofloxacin (32%)	Azithromycin (100%)	Amoxicillin (98%)	Co-amoxiclav (98%)
	*P. aeruginosa*	Levofloxacin (70%)	Ciprofloxacin (29%)	-	-	-	-
	*K. pneumoniae*	Levofloxacin (67%)	Ciprofloxacin (35%)	Amoxicillin (2%)	Co-amoxiclav (100%)	Amoxicillin (98%)	Azithromycin (98%)
Marie et al., 2010 [50]	-	-	-	-	-	-	-

**Table 3:** Quality assessment of studies.

	Selection				Comparability	Outcomes		
Reference	Representative of Sample ^A^	Sample Size ^B^	Non-Respondents ^C^	Ascertainment of Exposure ^D^	Comparability of Cohort Studies on Basis of Design ^E^	Assessment of Outcomes ^F^	Statistical Analysis ^G^	Quality Score
Batool et al., 2021 [23]	*	*	-	**	*	**	-	7
Balkhy et al., 2014 [24]	*	*	-	**	*	**	*	8
Farahat et al., 2021 [25]	*	*	-	*	*	**	*	7
Rahman et al., 2000 [26]	*	*	-	**	*	**	*	8
Memish et al., 2004 [27]	*	*	-	**	*	**	*	8
Alshahwan et al., 2019 [28]	*	*	-	*	*	**	*	7
Akbar et al., 2001 For CAP [29]	*	*	-	**	*	**	*	8
Babay et al., 2000 [30]	*	*	-	**	*	**	-	7
Albarrak et al., 2018 [31]	*	*	-	*	*	**	*	7
Obeid et al., 2015 [32]	*	*	-	**	*	**	-	7
Almuneef et al., 2004 [33]	*	*	-	*	*	**	*	7
Saleem et al., 2022 [34]	*	*	-	**	*	**	*	8
Al-Saed et al., 2013 [35]	*	*	-	*	*	**	*	7
Osman et al., 2020 [36]	*	*	-	*	*	**	*	7
Kabrah et al., 2018 [37]	*	*	-	**	*	**	*	8
Ibrahim et al., 2018 [38]	*	*	-	**	*	**	*	8
Al-Dorzi et al., 2012 [39]	*	*	-	*	*	**	*	7
Saleem et al., 2023 [40]	*	*	-	**	*	**	*	8
Othman et al., 2017 [41]	*	*	-	*	*	**	*	7
Balkhy et al., 2006 [42]	*	*	-	*	*	**	*	7
Al-Johani et al., 2010 [43]	*	*	-	**	*	**	*	8
Mwanri et al., 2014 [44]	*	*	-	*	*	**	*	7
Bshabshe et al., 2016 [45]	*	*	-	**	*	**	*	8
Zaki at al., 2021 [46]	*	*	-	*	*	**	*	7
Hakami et al., 2022 [47]	*	*	-	**	*	**	*	8
Walid et al., 2016 [48]	*	*	-	**	*	**	*	8
Al Munjem et al., 2022 [49]	*	*	-	**	*	**	*	8
Marie et al., 2010 [50]	*	*	-	*	*	**	-	6

A: * = truly representative or somewhat representative of average in target population; B: * = drawn from the same community; C: * = secured record or structured review; D: * = yes, - = no; E: * = study controls for age, gender, and other factors; F: * = record linkage or blind assessment; ** = both; G: * = follow-up of all subjects.

## Data Availability

Not applicable.
